# Emerging Role of the DREAM Complex in Cancer and Therapeutic Opportunities

**DOI:** 10.3390/ijms26010322

**Published:** 2025-01-01

**Authors:** Ye-Jin Hwang, Moon Jong Kim

**Affiliations:** 1 Department of Life Science, Gachon University, Seongnam 13120, Republic of Korea; hyjj1024@gachon.ac.kr; 2 Department of Health Science and Technology, GAIHST, Lee Gil Ya Cancer and Diabetes Institute, Incheon 21999, Republic of Korea

**Keywords:** DREAM complex, cancer cell quiescence, cell cycle regulation, cancer therapy, targeted therapy

## Abstract

The DREAM (dimerization partner, RB-like, E2F, and multi-vulval class B) complex is an evolutionarily conserved transcriptional repression complex that coordinates nearly one thousand target genes, primarily associated with the cell cycle processes. The formation of the DREAM complex consequently inhibits cell cycle progression and induces cellular quiescence. Given its unique role in cell cycle control, the DREAM complex has gained significant interest across various physiological and pathological contexts, particularly in conditions marked by dysregulated cell cycles, such as cancer. However, the specific cancer types most significantly affected by alterations in the DREAM complex are yet to be determined. Moreover, the possibility of restoring or pharmacologically targeting the DREAM complex as a therapeutic intervention against cancer remains a relatively unexplored area of research and is currently under active investigation. In this review, we provide an overview of the latest advances in understanding the DREAM complex, focusing on its role in cancer. We also explore strategies for targeting the DREAM complex as a potential approach for cancer therapeutics. Advances in understanding the precise role of the DREAM complex in cancer, combined with ongoing efforts to develop targeted therapies, may pave the way for new options in cancer therapy.

## 1. Introduction

The cell cycle comprises a series of tightly regulated fundamental processes by which a cell divides to produce two daughter cells. When the strict controls of the cell cycle are dysregulated, it can lead to abnormal conditions, such as the uncontrolled cell proliferation frequently observed in cancer [1,2]. Dozens of regulatory checkpoints have been identified and well characterized, including those at the G1-S and S-G2/M phase transitions [2,3]. However, among various transition points in the cell cycle, the regulatory signals and mechanisms governing the transitions from the quiescent G0 to the G1 phase or from the G2 to the G0 phase remain poorly understood. One complex extensively studied in this context is the DREAM complex, which uniquely induces and maintains cellular quiescence in the G0 state [4,5,6].

The DREAM complex is an evolutionarily conserved multi-protein complex found across species, from Caenorhabditis elegans (*C. elegans*) to humans; it even extends beyond the animals, with similar conservation observed in plants [4,5]. Initially, this complex was identified through the discovery of synthetic multi-vulval class B (synMuvB) proteins, including Rb-like *rbf* (*lin-35*), E2F-like *efl-1*, and DP-like *dpl-1*, along with unknown components such as *lin-9*, *lin-37*, *lin-52*, *lin-53* (RBBP4-like), and *lin–54*, all of which are associated with the multi-vulva phenotype in *C. elegans* [7,8,9]. Later, a similar complex containing RB-like *dRbfs* (*dRbf1* and *dRbf2*), E2F-like *dE2Fs*, and DP-like *dDP* was identified in *Drosophila*. Subsequent studies revealed that Rb/E2Fs/DP proteins form a larger complex with a sub-compartment called the MuvB core, composed of *Mip130* (*Lin-9*), *Mip40* (*Lin-37*), *Lin-52*, *Caf* (*RBBP4*), and *Mip 120* (*Lin-54*). Through these studies, the main structure of the DREAM complex was determined, as represented in *C. elegans* (DRM: DP-RB-MuvB) [10] and in *Drosophila* (dREAM) [11,12]. Soon after, homologs of the dREAM-like complex were also identified in mammalian cells (DREAM) and plants (Myb-MuvB) [13]. Importantly, while the composition of the DREAM complex varies slightly across evolutionary stages and species, it generally functions as a transcriptional repressor, binding to promoter regions and primarily acting to inhibit target gene expression [5,12,14,15].

**Figure 1:**
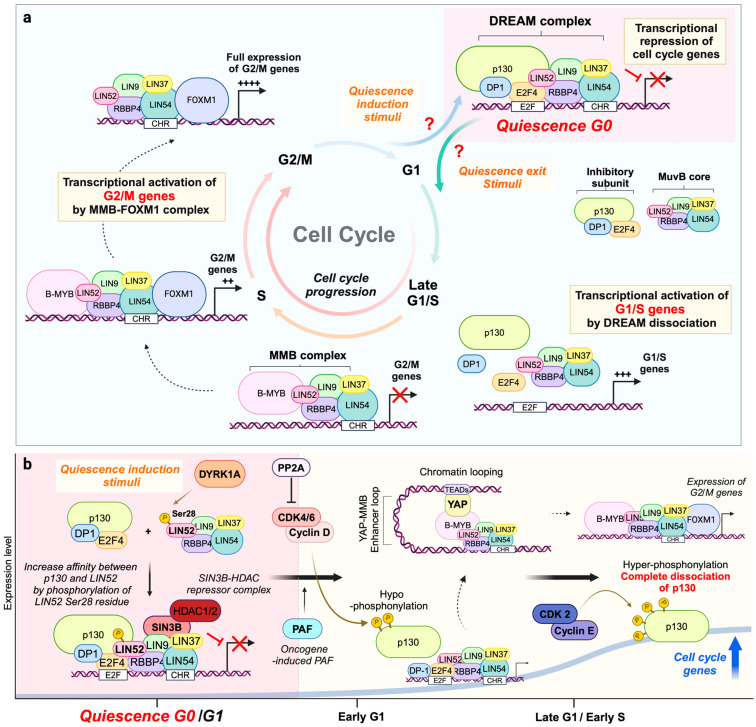
DREAM complex and its regulatory mechanism. (**a**) Transition of the DREAM complex during the cell cycle: The DREAM complex is composed of two parts: the transcriptional inhibitory module (comprising DP1, p130, and E2F4) and the multi-vulval class B core (MuvB core; comprising LIN9, LIN37, LIN52, LIN54, and RBBP4). In the G0/G1 phase, the DREAM complex is assembled, transcriptionally inhibiting its target genes, which results in cellular quiescence (G0). As the cell progresses through the cycle, the DREAM complex transitions into the MMB (MYB-MuvB) complex during G1/S and subsequently into the MMB-FOXM1 complex during the S/G2/M phases. These transitions activate the transcription of G1/S and G2/M genes previously repressed by the DREAM complex. Gene expression strengths are indicated as follows: ++, +++, ++++. (**b**) Representative regulatory mechanisms of the DREAM complex during the cell cycle: In the G0-G1 state, the phosphorylation of serine 28 on LIN52 by DYRK1A promotes the binding of p130 to the MuvB core, facilitating DREAM assembly. SIN3B-HDAC acts as a co-repressor during this process. The oncogenic factor PAF competes with p130 to bind to the MuvB core, promoting the G1/S transition. In early G1, the CDK4/6-cyclin D complex is activated, inducing the hypo-phosphorylation of p130. As the cell transitions from the G1 to the S phase, increased activity of CDK2 and cyclin E leads to the hyper-phosphorylation and inactivation of p130. During the G2/M phase, BMYB (a component of the MMB complex) interacts with YAP, a distant enhancer factor, to form enhancer loops that upregulate the expression of G2/M-specific genes. In the G2/M phase, PP2A acts as a key phosphatase against CDKs, a process essential to reset the cell to the G0 state.

In mammalian systems, p130 immunoprecipitation (IP) and mass spectrometry (mass-spec) analysis have provided detailed insights into the overall compartments of the DREAM complex [16,17,18]. The mammalian DREAM complex consists of several key proteins: E2F (E2F4 or E2F5), RB-like proteins (RB-like protein 2: RBL2/p130 or RB-like protein 1: RBL1/p107), their dimerization partners (DP-1 and DP-2), and the MuvB core, a sub-complex made up of LIN9, LIN37, LIN52, LIN54, and RBBP4 (Figure 1a). Functionally, the DREAM complex is divided into two subgroups: the transcriptional inhibitory module (DP1, p130, and E2F4) and the MuvB core [8,9,10]. The dissociation of inhibitory subunits from the DREAM complex induces its disassembly and the subsequent activation of target genes. In mammals, p107 and p130 belong to the retinoblastoma (Rb) protein family, which includes RB1 (Rb), p107 (RBL1), and p130 (RBL2). Like RB1, these pocket proteins inhibit E2F transcription factors through direct protein–protein interactions. Among the eight E2F family proteins, RB-regulated E2F1, E2F2, and E2F3a act as transcriptional activators, while the remaining E2Fs serve as transcriptional repressors [18,19,20,21,22,23]. Within the DREAM complex, repressive E2F4 and E2F5 interaction with p130 and p107 is preferentially observed in IP and proteomic analysis. Notably, p130 is expressed robustly in the G0/early G1 phase, suggesting that the DREAM complex in this phase is primarily mediated by p130. Moreover, IP analysis shows that p130 predominates in the DREAM complex during G0/G1. Unlike p130, p107 is a transcriptional target of the DREAM complex. It appears to play a primary role in late G1 and S phases, after the p130-containing DREAM complex dissociates due to the decreased p130 levels brought about by the regulation of phosphorylation [16,18].

The mammalian DREAM complex has been recognized as a transcriptional coordinator of G0/G1 cell cycle regulation and cellular quiescence, as further described in a later section of this review. Additionally, recent studies have revealed that the DREAM complex participates in a range of cellular processes, including DNA repair, chromatin remodeling, senescence, and stem cell regulation beyond the regulation of cell cycles [4].

Given its pivotal function in controlling the cell cycle and promoting cellular quiescence, the DREAM complex has become a major focus in cancer research [4,5]. Researchers hypothesize that the disruption of the DREAM complex may serve as a key indicator of cancer progression. However, the extent of DREAM complex disruption remains largely unknown, as do the specific cancer types associated with its deregulation. Additionally, the potential for restoring or pharmacologically modulating the DREAM complex as a therapeutic strategy in cancer is being explored in early studies, but still requires further investigation [4,24,25].

## 2. The DREAM Complex in Cell Cycle Regulation and Quiescence

The formation of the DREAM complex changes dynamically depending on the cell cycle stage and physiological conditions. When assembled, the DREAM complex binds to the promoters of target genes and transcriptionally suppresses them [16,17,26,27,28,29,30] (Figure 1a). Chromatin immunoprecipitation (ChIP) sequencing analyses have shown that DREAM complex components—p130, E2F4, LIN9, and LIN54—bind to promoter regions near the transcription start site, with a stronger binding affinity in G0 than in the other cell cycle phases [16]. Studies have further revealed that the DREAM complex binds to target promoters either through the conventional E2F DNA-binding motifs recognized by E2F4 or via the cell cycle gene homology region (CHR) motif recognized by LIN54 [16,31,32,33,34,35]. Through these interactions, the DREAM complex represses the expression of approximately 1000 genes involved in cell cycle progression, DNA synthesis, DNA repair, mitosis/chromosomal segregation, and histone modification [16,36,37,38,39]. Consequently, the formation of the DREAM complex induces cell cycle arrest in the G0/G1 phase, leading to cellular quiescence [4,5].

An intriguing aspect of the DREAM complex is the dual functionality of its MuvB core subunit. During the G0/G1 phase, the MuvB core promotes quiescence by associating with inhibitory subunits to form the DREAM complex (Figure 1a; G0-G1). Conversely, in the S/G2/M phases, it transitions into a transcriptional activator by recruiting two key transcriptional activators, BMYB (Myb-related protein B, also known as MYBL2) and Forkhead box M1 (FOXM1) [29] (Figure 1a; S-G2/M). Together, these form the MMB (MYB-MuvB)–FOXM1 complex, which functions as a mitotic transcriptional activator, upregulating the expression of cell cycle genes previously suppressed by the DREAM complex during the G0/G1 phase. The transition cue and processes governing this transition are not yet fully understood, but it is believed to begin with the dissociation of DP1, p130, and E2F4 from the DREAM complex. Following this dissociation, the MuvB core recruits BMYB in the G1/S phase and subsequently recruits FOXM1 in the S/G2/M phase (Figure 1a; S-G2/M). This shift transforms the DREAM complex into the MMB complex in G1/S and then the MMB–FOXM1 complex in the S/G2/M phase. This transition from a repressive to an activating complex promotes the transcription of cell cycle genes, thereby driving the cell cycle through the S/G2/M phases.

Importantly, the regulation of the cell cycle by the DREAM complex is mediated by its interactions with various other cell cycle regulators. For example, the DREAM complex and the MMB complex primarily regulate two categories of cell cycle genes: those involved in the G1/S transition and those governing the G2/M transition [4,6,27] (Figure 1a). Notably, the G1/S transition checkpoint is predominantly regulated by RB-E2F1, a member of the same pocket protein family, while the DREAM complex plays additional, complementary roles. ChIP and transcriptome analyses have revealed that the DREAM complex cooperates in G1/S transcription by simultaneously regulating a subset of genes controlled by RB-E2F1, including *CCNE*, *BMYB*, and *CDK2* [4,6,16,27,40,41]. While the DREAM complex does regulate a subset of G1/S genes controlled by RB-E2F1, its regulatory scope is somewhat distinct and does not fully compensate for the broader range of genes governed by RB [6,42,43]. In contrast to RB-E2F1, the DREAM complex has a pivotal role in the transcriptional regulation of G2/M genes [4,6]. In the G2/M transition, the DREAM complex is essential for the full expression of hundreds of G2/M phase genes [4,6,18,29,44]. However, the regulation of these genes is not solely dependent on the DREAM complex but is also influenced by interactions with various cell cycle-dependent cyclin/CDK complexes and combinations of transcriptional factors [4,6,30,45,46,47,48,49,50].

Additionally, key cell cycle regulators, such as p53, share multiple overlapping targets with the DREAM complex, simultaneously influencing the regulation of these genes [36,37,39,42]. Moreover, the activity of the DREAM complex is further fine-tuned through interactions with CDK inhibitors (e.g., p21, p27, p57) and regulatory kinases, forming a tightly coordinated network that ensures precise cell cycle control [4,6,37,51].

## 3. Regulatory Mechanisms of the DREAM Complex

Despite extensive research, the regulatory signaling and mechanisms of the DREAM complex remain largely unknown and require further investigation. The currently identified regulatory mechanisms are outlined below (Figure 1b).

### 3.1. DYRK1A Mediates DREAM Complex Assembly

The primary mechanism that enhances the affinity between p130 and MuvB for DREAM complex formation is the phosphorylation of the 28th serine residue (Ser28) of LIN52 by DYRK1A and DYRK1B (dual-specificity tyrosine-regulated kinases 1A and 1B) [27] (Figure 1b). The DYRK family is characterized as a proline-directed kinase, utilizing the RPX(S/T)P consensus sequence to phosphorylate target proteins [52]. Using multidimensional protein identification technology (MudPIT, also known as shotgun proteomics), Larisa et al. discovered that the MuvB complex incorporated by Ser28-phosphorylated LIN52 predominantly binds to p130 and E2F4. They also identified evolutionarily conserved target sequences for DYRK1A and DYRK1B in the region surrounding the Ser28 residue of LIN52 and demonstrated that these kinases co-immunoprecipitated with LIN52 and directly phosphorylated Ser28 [16].

It has been reported that the LxCxE motif plays a crucial role in binding to the RB protein family by interacting with the pocket cleft [53,54]. Viral oncoproteins—such as high-risk human papillomavirus (HPV) E7, SV40 Large T antigen, and adenovirus E1, all of which can lead to cancer—utilize this motif to inactivate RB proteins [55,56]. Importantly, LIN52 contains an evolutionarily conserved non-canonical LxSxE motif. The substitution of the serine residue in this motif with cysteine (LIN52-S20C) enhances its binding to p130 and inhibits cell proliferation [27]. These findings suggest that two types of interactions exist between p130 and LIN52: a weak interaction mediated by the LxSxE motif and a regulatory interaction that strengthens p130–LIN52 binding, induced by the phosphorylation of Ser28 in LIN52 and mediated by DYRK1A.

The mechanism by which DYRK1A regulates DREAM complex assembly is reported to function conservatively across various processes, including cancer, cellular aging, and regeneration [4,25,27,57].

### 3.2. CDKs and Cyclins Disassemble the DREAM Complex for Cell Cycle Progression

Cyclin-dependent kinases (CDKs), together with their cyclin partners, are serine/threonine protein kinases that disassemble the DREAM complex and facilitate cell cycle progression by regulating the phosphorylation of p130 [30,58,59,60,61,62] (Figure 1b). As is well documented, CDK–cyclins regulate RB activity through phosphorylation, causing RB to dissociate from E2F and activate transcriptional regulation. Similarly, p130 (RBL2) follows a comparable mechanism [45,46,47,48,49,50]. In its non-phosphorylated form, p130 binds to E2F4 and other component proteins to form the DREAM complex, suppressing the expression of target genes. In contrast, the phosphorylation of p130 causes its dissociation from E2F4, leading to its degradation, reduced protein stability, and DREAM complex disassembly.

This regulatory process of p130 phosphorylation occurs sequentially through the combined action of two CDK–cyclin pairs [4,6,30]. In the early G1 phase, the CDK4/6–cyclin D complex phosphorylates p130. Among the approximately 22 phosphorylation sites on p130, three specific sites—S672 and S1035 at the C-terminus and T401 at the N-terminus—have been identified as targets of CDK4/6–cyclin D [61]. This hypo-phosphorylation decreases p130’s affinity for the MuvB core, allowing cells to exit the G0 phase, progress through early G1, and advance in the cell cycle. As cells transition from the G1 to the S phase, the CDK2–cyclin E complex induces the hyper-phosphorylation of p130 [58,59,60,62]. This hyper-phosphorylation is thought to inactivate the DREAM complex completely and target p130 for proteasome-mediated degradation. Consequently, the MuvB core transitions into the MMB complex by associating with BMYB and FOXM1, promoting cell cycle progression.

Notably, we poorly understand the processes following the hypo- and hyper-phosphorylation of p130 in its role in the cell cycle. Further investigation is needed to elucidate how p130’s localization changes, the mechanisms governing its nuclear export, and the proteins involved in its degradation.

### 3.3. Epigenetic Regulation of the DREAM Complex

As DREAM is a transcriptional repressor complex, a research field is emerging on its interactions with co-transcriptional factors, repressors, and epigenetic regulators. Recently, one such epigenetic regulator was identified through mass-spec analysis [38]. A component of the transcriptional repressor complex, Sin3B, recruits histone deacetylases (HDACs) to modify the histones, resulting in a dense chromatin structure and repression of target gene expression. In mass-spec analysis using Sin3B, several DREAM complex components were identified as binding partners of the Sin3B–HDAC complex in T98G glioblastoma cells. Further studies revealed that the DREAM complex represses target genes through interactions with the Sin3B–HDAC complex, crucial for maintaining cellular quiescence.

Recent research has also uncovered additional epigenetic regulation of the DREAM complex. YAP (Yes-associated protein), a transcriptional co-activator, and its partner TEADs, are known to play an oncogenic role by promoting cell cycle progression and increasing resistance to chemotherapy [63,64,65,66]. One study demonstrated that YAP physically interacts with BMYB, acting as a distant enhancer to promote G2/M gene expression [66]. Also, other studies have revealed that the YAP/TEAD binding enhancer forms a chromatin loop with the BMYB/MMB complex, transcriptionally upregulating the expression of target genes in the contexts of lung and breast cancer [66,67]. Subsequent studies of YAP’s enhancer-binding role were conducted in human breast epithelial cells, showing an increase in RNA polymerase II with serine 5 phosphorylation at common target gene loci, such as CDC20, TOP2A, and NEK2, which are also targets of both YAP and the MMB complex [67]. These findings provide further insight into how the chromatin structure regulates the activity of the DREAM complex during cell cycle regulation in cancer cells.

### 3.4. Other Identified DREAM Regulators: GSK3, PP2A, and PAF

Of the remaining non-CDK-mediated phosphorylation sites, p130, S948, S982, and S962 have been identified as phosphorylation targets of glycogen synthase kinase 3 (GSK3) [1,22]. Similar to DYRK1A, GSK3 is a well-known proline-directed kinase, and these phosphorylation sites are located in a specific loop region of the p130 protein that is absent from other RB family proteins. Interestingly, GSK3-mediated phosphorylation is suggested to stabilize p130 during the G0 phase, in contrast to the CDK–cyclin complex, which induces p130 instability [22,61]. However, further research is needed to understand how these phosphorylation events integrate with existing regulators and their physiological and pathological significance [22].

Protein phosphatase 2A (PP2A) is a heterotrimeric protein phosphatase accounting for a broad range of phosphatase activities and is widely considered a tumor suppressor [68]. Interestingly, a study reported that PP2A activity during the G2 phase is essential for DREAM complex formation in the next G0/G1 phase [58,69,70,71]. Nana et al. demonstrated that inhibiting PP2A in the G2 phase induces an overexpression of cyclin E, which disrupts quiescence. Moreover, this study suggested that the PP2A-mediated repression of Ras signaling plays a critical role in maintaining stable cell cycle arrest during the G0 phase [69]. Consistent with this was the finding that treatment with the phosphatase inhibitor okadaic acid disrupted DREAM complex formation in the subsequent G1 phase.

Recently, PAF (also known as PCLAF/KIAA0101) was identified as another regulator of DREAM complex assembly. PAF is a 111-amino-acid nuclear protein reported to promote cell cycle progression in various contexts, including multiple cancers [57,72]. Kim et al. discovered that PAF interacts with RBBP4, inducing the dissociation of p130 and facilitating the transition of the DREAM complex to the MMB–BMYB complex during lung tumorigenesis [57]. Using an in vivo GEMM lung tumor mouse model, their study highlighted that DREAM complex disassembly is a prerequisite for tumorigenesis. Additionally, they demonstrated that this process could be reversed by DREAM-inducing compounds identified through drug-repurposing screening.

The precise mechanisms and upstream signals governing the DREAM complex remain unclear. However, ongoing active research on the DREAM complex across various contexts is expected to reveal additional regulatory mechanisms soon.

## 4. Physiological and Pathological Roles of the DREAM Complex

**Figure 2:**
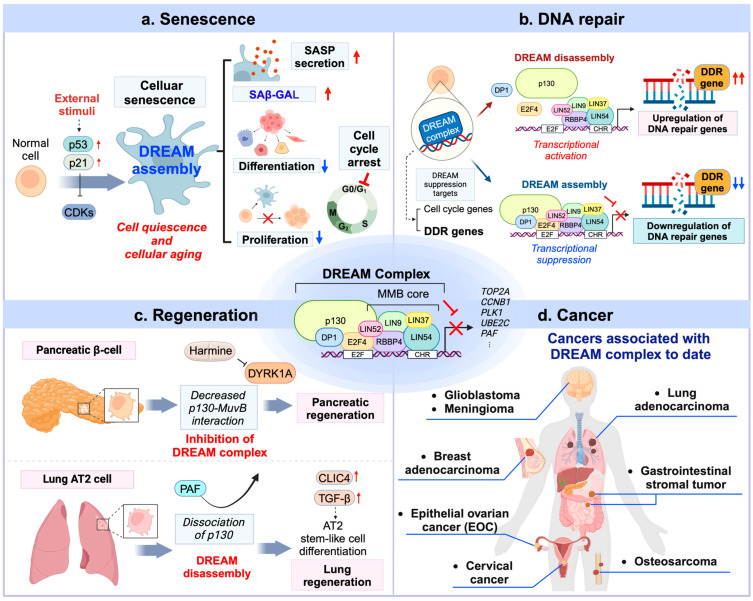
Physiological and pathological roles of the DREAM complex. (**a**) DREAM complex and cellular senescence. The formation of the DREAM complex induces cell quiescence and promotes cellular senescence. In this state, the cell cycle is arrested, cellular proliferation is impaired, and differentiation is restricted. Key characteristics of senescence include cell enlargement, secretion of the senescence-associated secretory phenotype (SASP), and an increase in SAβ-gal, a well-known marker of senescence. (**b**) DNA repair role of the DREAM complex. The DREAM complex regulates DNA damage response (DDR) genes. When the DREAM complex is assembled, these DDR-associated genes are repressed, suppressing DDR activity. Conversely, the disassembly of the DREAM complex activates DDR, enabling cells to respond to DNA damage. (**c**) Role of the DREAM complex in regeneration. Inhibition of the DREAM complex, such as by Harmine, has been shown to promote regeneration in pancreatic β cells. Additionally, disassembly of the DREAM complex by PAF facilitates lung regeneration by promoting the differentiation of alveolar type 2 (AT2) cells. (**d**) DREAM complex-related cancers to date. Cancers associated with the DREAM complex, as reported in recent studies, are depicted in the figure.

Given its pivotal role in regulating cell cycle and cellular quiescence, the DREAM complex has been extensively studied in various physiological and pathological contexts, including senescence, DNA repair, tissue regeneration, and cancer (Figure 2). Below, we provide a brief overview of key physiological and pathological phenomena associated with the DREAM complex, including recent discoveries. Research related to cancer will be discussed in more detail in a later section.

### 4.1. Role of the DREAM Complex in Cellular Senescence

Cellular senescence causes cells to cease proliferating and renders them unresponsive to external stimuli [73]. This can be induced by external DNA damage, aging, or oncogenes and serves as a complex cellular defense mechanism linked to various diseases [73,74]. The connection between the DREAM complex and senescence first emerged when its role in Ras-oncogene-induced senescence (OIS) was reported [27]. Larisa et al. demonstrated that the disruption of DREAM complex assembly interfered with Ras-induced senescence in HRAS-G12V-expressing BJ-hTERT fibroblasts. Notably, cells expressing the LIN52-S28A mutant or treated with shRNA targeting DYRK1A exhibited significantly reduced senescence-associated β-galactosidase (SAβ-gal) activity and increased proliferation in response to HRAS-G12V compared to controls.

In addition, a recent study has linked genotoxic stress to cellular senescence through the p21CIP1–CDK4–DREAM axis [51] (Figure 2a). During genotoxic stress, p53-p21CIP signaling inhibits CDK4, thereby promoting DREAM complex formation. This results in a suppressed DNA damage response (DDR), increased DNA damage accumulation, and, ultimately, the induction of cellular senescence across various normal cell lines.

### 4.2. Role of the DREAM Complex in DNA Repair

The DREAM complex is expected to be involved in DNA repair processes, as the hundreds of DNA repair genes active during the S phase are mostly its repression targets. Recent studies have begun to uncover these connections and underlying mechanisms.

One key connection was identified using LIN37 knockout (KO) cells. In pre-B cells and mammary epithelial cells [75], Chen et al. demonstrated that the loss of LIN37–DREAM function leads to an increase in homologous recombination (HR) proteins. Further investigations revealed that the LIN37–DREAM complex specifically reduces unnecessary DNA end resection and HR activity during the G0 quiescent state. This study provided the first evidence linking the DREAM complex to DDR under genotoxic stress.

In *Arabidopsis*, an evolutionarily conserved homolog, LIN37B, cooperatively functions as part of the DREAM complex with the retinoblastoma homolog RBR1 [76]. Similar to its mammalian counterpart, the LIN37B-containing DREAM complex in *Arabidopsis* responds to DNA damage by inducing cell cycle arrest and repressing root growth [76]. These findings highlight the evolutionary conservation of DREAM complex-mediated DNA repair functions, extending beyond animal species.

More recently, studies in *C. elegans* revealed that over half of the DDR genes contain cell cycle-dependent element (CDE) and CHR promoters, which are repressed by the DREAM repressor complex [77] (Figure 2b). Intriguingly, loss-of-function mutations in DREAM complex components in worms resulted in enhanced DDR activity and increased resistance to various forms of DNA damage. Similar results were confirmed in mammalian systems. In U2OS cells, the dissociation of the DREAM complex induced by DYRK1A inhibitors, such as Harmine and INDY, increased resistance to DNA damage-induced cell death. Furthermore, treatment with these inhibitors in *Ercc1*-deficient mice reduced retinal damage, further supporting the DREAM complex as a conserved master regulator of DDR across animal species.

These recent updates emphasize the therapeutic potential of targeting the DREAM complex to enhance DNA repair and mitigate damage-related pathologies. Furthermore, the regulatory mechanisms underlying the DREAM complex in DNA repair are a highly active research area. A key focus for future studies may be to explore its mechanistic roles and potential applications, particularly in cancer and cancer therapy, aligning with the objectives of this review.

### 4.3. Role of the DREAM Complex in Regeneration

Recent reports have emphasized the role of the DREAM complex in regulating the regeneration process and stem cell activity. Wang et al. investigated how DYRK1A inhibitors promote the regeneration of pancreatic beta cells and found that DYRK1A facilitates this regeneration by inhibiting DREAM complex formation [44] (Figure 2c). Furthermore, Kim et al. demonstrated that the disassembly of the DREAM complex by PAF promotes the differentiation of AT2 stem-like cells by enhancing downstream CLIC4–TGFβ signaling during lung regeneration [78] (Figure 2c). These studies are significant as they elucidate the role of the DREAM complex in regulating quiescent cell activity and regeneration. It is expected that further research will soon uncover additional insights into the biological role of the DREAM complex in quiescent stem cells.

## 5. The Emerging Role of the DREAM Complex in Cancer

Since abnormal and uncontrolled cell proliferation is a hallmark of cancer, there is an inevitable link to the DREAM complex, which governs cell quiescence and cell cycle arrest. Indeed, several genes—such as BMYB, FOXM1, polo-like kinase 1 (PLK1), Aurora kinase A (AURKA), and CCNB1—regulated and suppressed by the DREAM complex are overexpressed in various cancers and are associated with a poor cancer prognosis [79,80,81,82,83,84,85,86]. These genes also contribute to bypassing cell cycle arrest and function as drivers of tumorigenesis. Although research on the DREAM complex in cancer is continually expanding, much remains unclear. In particular, it is uncertain whether the DREAM complex plays a significant role in specific types of cancers or across all cancer. Furthermore, it is unclear whether changes in the DREAM complex actually affect patient survival and cancer progression in certain cancer types. To date, DREAM-associated functions have been reported sporadically in a few cancer types (Figure 2d). We listed putative or confirmed DREAM-associated cancers in Table 1.

The association between the DREAM complex and cancer was first reported in gastrointestinal stromal tumors (GISTs) [24]. Sergei et al. demonstrated that imatinib treatment induces quiescence in GIST cancer cells, a process mediated by the DREAM complex. Disrupting the DREAM complex using siDYRK1A and siLIN52 combined with imatinib treatment maximized imatinib’s efficacy and significantly enhanced cell death and apoptosis. This finding is meaningful as it highlights the potential of targeting the DREAM complex as a novel approach to cancer therapy.

Direct links between DREAM complex dysregulation and cancer have also been observed in virus-infected cancers, such as cervical and ovarian cancers. HPV infection is a well-known cause of cancer and disrupts DREAM complex formation. In cervical cancer, the E7 viral protein uses the LxCxE motif to interact with BMYB and FOXM1, destabilizing p130 and promoting MMB complex activity, which increases G2/M gene expression and supports cell cycle progression [55,88]. In epithelial ovarian cancer, inhibiting DYRK1A via siRNA knockdown or CDK inhibition compromised the DREAM complex and enhanced the viability of ovarian cancer cell spheroids [25].

Interestingly, most of the regulatory mechanisms, such as the structural assembly of the DREAM complex, subunit disassembly, and BMYB regulation of the G1/S transition process, have been studied in T98G cells, a glioblastoma cell line derived from brain tumors [16,27,91]. Recent studies further support this DREAM mechanism as they show its significant role in meningiomas, the most common type of brain tumor [89]. In type C meningiomas, which have the highest recurrence rate, the DREAM complex loses its inhibitory function, and the expression of FOXM1 and BMYB is simultaneously increased.

Additionally, colorectal cancer (CRC) cells, such as HCT116, have frequently been used as a model to validate DREAM complex mechanisms [36,42], suggesting DREAM’s potential involvement in CRC tumorigenesis, though the clinical implications remain underexplored. More recently, in lung adenocarcinoma, the nuclear protein PAF was identified as a dissociation factor of the DREAM complex [57]. Oncogene-induced PAF binds to RBBP4, disrupting the DREAM complex and promoting cell cycle progression and tumorigenesis. Collectively, these findings indicate that the DREAM complex may be involved in various cancer types.

Many studies have consistently linked the inhibition or disruption of the DREAM complex to the development and proliferation of certain cancers. Notably, genetic alterations in DREAM complex-associated genes are rarely reported across cancers. Wang et al. reported that an analysis of 33 TCGA-based cancers showed an overall DREAM complex mutation rate of approximately 10% [92], with higher mutation rates only in a few cancers such as uterine corpus endometrial cancer, skin cutaneous melanoma, kidney renal clear cell carcinoma, and lung squamous cell carcinoma. Given that the DREAM complex consists of approximately nine components, the overall mutation rate is not particularly high—in fact, it is similar to those of other passenger mutations found in cancers—contrasting with the higher rates of mutations observed in key driver oncogenes and tumor suppressor genes. These findings suggest that DREAM complex deregulation in cancers primarily occurs through a range of post-translational modifications rather than genetic mutations.

## 6. Targeting the DREAM Complex for Cancer Therapy

Given the influence of the DREAM complex on the cancer cell cycle, it has emerged as a promising therapeutic target, and there have been proposals that it may be effectively combined with various anticancer treatments. Additionally, the role of the DREAM complex in maintaining cancer cell quiescence has attracted attention to its potential as a target for eliminating residual quiescent cancer cells that might persist in a dormant state and exhibit resistance after initial treatment [93,94,95]. However, research on such combination therapies remains limited, and the involvement of the DREAM complex in scenarios of minimal residual cancer cells has yet to be explored [95].

Recent studies have suggested that the “inhibition of DREAM complex formation” in certain cancer types may enhance anticancer effects by reactivating quiescent cancer cells, such as through DYRK1A inhibitor treatment [24,25,57,77,88]. Conversely, recent drug repurposing efforts have identified two compounds that appear to promote DREAM complex formation [57]. While the exact mechanisms are uncertain, these chemicals suggest that the “induction of the DREAM complex” could also feasible strategy.

**Figure 3:**
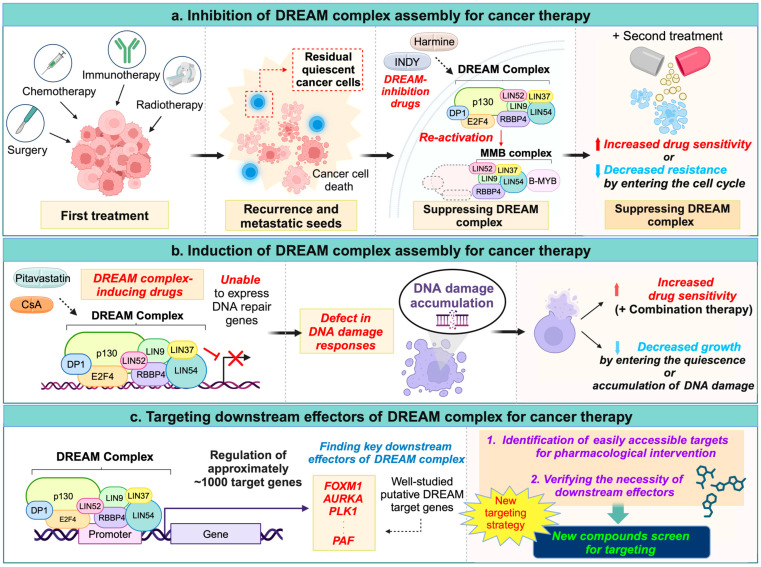
Schematic representation of DREAM complex-targeting strategies for cancer therapy. (**a**) Inhibition of DREAM complex assembly: This strategy involves disrupting the assembly of the DREAM complex as part of a secondary treatment. By “waking up” residual microscopic cancer cells that remain quiescent after the initial therapy, these cells are made responsive to subsequent treatments. This approach is particularly effective when genes suppressed by the DREAM complex contribute to drug resistance. (**b**) Induction of DREAM complex assembly: Promoting the assembly of the DREAM complex suppresses key gene sets that provide specific survival advantages to cancer cells, such as those involved in DNA repair. This suppression sensitizes cancer cells to therapeutic agents, enhancing their efficacy. (**c**) Targeting downstream effectors of the DREAM complex: This approach focuses on identifying critical downstream genes regulated by the DREAM complex that are easily accessible for drug discovery. By targeting these genes through pharmacological interventions, similar therapeutic effects can be achieved as with direct DREAM complex targeting.

Importantly, the rarity of genetic mutations in the DREAM complex [92] raises the value of targeting this complex as a cancer therapy option, in contrast to tumor suppressors like p53, PTEN, and RB, which are frequently mutated and thus challenging to target effectively [96]. In this approach, drugs that either inhibit or promote DREAM complex formation could be combined with existing anticancer therapies, offering various strategies for cancer treatment, which are discussed below (Figure 3).

### 6.1. Inhibition of the DREAM Complex Assembly for Cancer Therapy

Several studies indicate that blocking DREAM complex formation can enhance cancer cell sensitivity to chemotherapy [24,25]. This approach aligns with recent “cancer awakening” strategies highlighted in recent reviews [93,94]. Inhibiting the DREAM complex may allow for reactivating microscopic residual, drug-resistant cancer cells that persist after chemotherapy, so these cells become susceptible to subsequent anticancer treatments (Figure 3a).

Indeed, the depletion of DYRK1A phosphorylation by Harmine or INDY enhanced the response to carboplatin in EOC spheroid cells [25] Similarly, Harmine treatment of imatinib-induced gastrointestinal stromal cancer cells reduced quiescent cells and increased apoptosis by awakening the cell cycle [24].

However, it is important to note that DREAM inhibition does not consistently induce the sensitivity to chemotherapy across all cancer types. For instance, a recent study showed that Harmine treatment in osteosarcoma inhibits the DREAM complex, resulting in the upregulation of DNA repair genes and a reduction in DNA damage [77]. Moreover, it remains unclear which chemotherapeutic agents may synergize with DREAM complex inhibition. Additionally, the efficacy of this approach in fully eradicating residual cancer cells has not yet been experimentally validated. Further research will be crucial in advancing this promising therapeutic strategy.

### 6.2. Induction of the DREAM Complex Assembly for Cancer Therapy

Recent reviews have proposed a strategy for holding quiescent cancer cells in a dormant state to prevent recurrence [93,94,97]. For example, breast cancer patients who continued low-dose tamoxifen adjuvant therapy experienced a prolonged suppression of cancer recurrence [98]. This approach suggests that low doses of chemotherapeutic agents help maintain residual cancer cells in a quiescent state. If anticancer drugs like tamoxifen are well tolerated at low concentrations, extended treatment potentially prevents cancer recurrence and metastasis by keeping residual cancer cells dormant and suppressing their reactivation. Promoting DREAM complex formation may thus help maintain cancer cell quiescence (Figure 3b).

Recent in vivo studies have shown that clinically available drugs, such as cyclosporin A (CsA) and pitavastatin, can promote DREAM complex formation and induce the quiescent phase in lung cancer cells and in vivo lung cancer models [57]. While the immunosuppressant CsA may not be suitable for long-term use, pitavastatin—a third-generation statin used for hyperlipidemia—has the potential for extended treatment [99,100]. Although further validation is required on this mechanism, the findings show significant promise.

Palbociclib is a selective inhibitor of cyclin-dependent kinases CDK4 and CDK6, originally designed to target RB regulation, which controls the G1/S cell cycle transition [101]. It has been reported to be effective in estrogen receptor positive (ER+) breast cancer when combined with existing anticancer drugs [101,102]. However, there is considerable variability in patient responses, and the exact mechanisms behind these variations have not been clearly understood until recently [103,104]. A recent important study, which utilized meta-data analysis and experiments with patient cohorts, revealed the underlying mechanisms responsible for the large variation in responses to CDK4/6 inhibitors [90]. Kudo et al. elegantly identified that p53 signaling deficiency was commonly observed in patient populations unresponsive to CDK4/6 inhibitors. Their experimental findings showed that this phenomenon resulted from a deficiency in the p53-p21 signaling axis, which led to CDK2 activation, subsequently inactivating the DREAM complex even in the presence of CDK4/6 inhibition. They demonstrated that the co-administration of CDK4/6 and CDK2 inhibitors improved treatment efficacy in p53-deficient breast cancer by restoring the DREAM complex, as confirmed by both preclinical studies and clinical samples. This study is particularly significant as it uncovers the mechanisms underlying CDK4/6 drug resistance and provides critical insights into the overlapping functions of RB and the DREAM complex, emphasizing the need to co-target these pathways for improved therapeutic outcomes. Further research is necessary to better understand this mechanism and identify the specific cancer types and mutations where CDK4/6 inhibitors will be most effective.

Based on long-standing concepts, DREAM complex inhibition was thought to silence cancer cells and contribute to drug resistance [4]. However, recent research suggests an alternative perspective: the DREAM complex is a master regulator of hundreds of DNA repair genes [77]. Since the DNA repair response is crucial for cancer cell survival and drug resistance during chemotherapy, inhibiting the DREAM complex in combination with chemotherapy may thus induce the synthetic lethality of cancer cells.

Notably, Bujarrabal-Dueso et al. found that the DREAM complex represses DNA repair genes, and the disruption of the DREAM complex state was shown to promote resistance to DNA damage [77]. When cells were treated with the DYRK1A inhibitor Harmine or INDY, which disrupts p130’s binding ability in forming DREAM complexes, they showed a reduced sensitivity to DNA damage. Meanwhile, DREAM complex-inducing agents, such as CsA and pitavastatin, have demonstrated anticancer-enhancing effects in various cancers when used in combination therapies, as observed in clinical trials. These findings suggest that strategies promoting DREAM complex formation could hold promise for cancer treatment. However, the underlying mechanisms remain unclear and are likely influenced by various targets. Therefore, further investigations are essential to elucidate the pharmacological mechanisms of these drugs and validate the corresponding hypotheses for advancing cancer therapy.

### 6.3. Targeting Downstream Effectors of the DREAM Complex for Cancer Therapy

The ideal approach to harness the DREAM complex for cancer treatment is to either promote or inhibit its formation. However, the regulatory mechanisms that control DREAM complex assembly remain poorly understood, and specific drug targets and mechanisms for these controls are largely unknown. Currently, the only drugs linked to DREAM complex modulation are DYRK1A inhibitors, along with CsA and pitavastatin. However, DYRK1A has numerous targets across different contexts, and the precise mechanisms by which CsA and pitavastatin affect the DREAM complex are still unclear.

If directly controlling the DREAM complex remains challenging, an alternative strategy could be to target key effectors among its downstream-regulated genes (Figure 3c). The DREAM complex regulates hundreds of genes, including druggable kinases and enzymes, which could serve as viable therapeutic targets. Notably, additional efforts are needed to validate whether these downstream genes are essential effectors in specific cancer types. Recent studies indicate that downstream genes of the DREAM complex, such as FOXM1, PLK1, and AURKA, are critical for cancer cell proliferation across various cancers [80,83,84,85,105]. Several compounds targeting these genes are currently in clinical trials, suggesting that the strategy of “targeting downstream effectors of the DREAM complex” could hold promise for cancer therapy (Figure 3c).

## 7. Conclusions and Perspectives

Recent studies and growing interest in the DREAM complex have led to the development of small chemicals designed to target this complex. However, several key questions remain unanswered: (1) How and which factors are involved in the deregulation of the DREAM complex in cancer? (2) Which cancers are most specifically affected by the DREAM complex? (3) Could targeting the DREAM complex indeed help treat cancer and improve patient survival?

Although the pharmacological mechanisms remain unclear, clinically used drugs targeting the DREAM complex—such as pitavastatin, CsA, and Harmine—show promise as potential therapeutic options for cancers influenced by DREAM complex activity. Additionally, several DREAM complex-regulated genes, including AURKA, PLK1, and FOXM1, are being investigated as potential treatment targets.

Taken together, we dream to see the development of new cancer treatment options in the near future, based on targeting this emerging mechanism, the DREAM complex.

## Figures and Tables

**Table 1:** Cancers associated with the DREAM complex and related research to date.

Cancer Type	Cell lines and In Vivo Models Used	Methods	DREAM-Modulating Chemicals	Clinical Relevance	Remarks	Ref.
Glioblastoma	T98G	- Proteomic analysis (MudPIT) - DREAM complex subunit co-immunoprecipitation - siRNA (RBL1/RBL2, LIN9, LIN54, and E2F4) - ChIP-seq (p130, E2F4, LIN9, and LIN54)	-	Not determined (N.D.)	Mammalian DREAM complex and its target genes were first discovered	[16]
		- Proteomic analysis (MudPIT) - Mutational analysis of LIN52-S28 and LIN37-S182 residues - shLIN52 and DYRK1A overexpression	Harmine *	N.D.	Identified DYRK1A-mediated DREAM assembly phosphorylation site (LIN52-S28)	[27]
Osteosarcoma	U2OS	RNA-seq after treatment with DYRK1A inhibitors	Harmine * INDY *	N.D.	DREAM complex inhibition boosted the DNA damage repair response, a mechanism conserved from *C. elegans* to humans	[77]
		- Proteomic analysis (MudPIT) - Mutational analysis of LIN52-S28 and LIN37-S182 residues - shLIN52 and DYRK1A overexpression	Harmine *	N.D.	Identified DYRK1A-mediated DREAM assembly phosphorylation site (LIN52-S28)	[27]
Gastro-intestinal stromal tumor	GIST882 cells and patient-derived xenografts (PDXs) from two patients bearing KIT p.V650D or KIT p.A502_Y503 dup mutations	- siRNA (DYRK1A and LIN52) - GIST882 xenograft - KIT-mutated PDXs	Harmine *	N.D., but in vitro experiments showed enhanced apoptosis with imatinib treatment	Proposed Harmine as a therapeutic target for imatinib-induced dormant gastrointestinal stromal tumors	[24]
Epithelial ovarian cancer (EOC)	Various EOC cell lines including iOvCa (129, 130, 147E2, 185, 246, and 256), OVCAR3, OVCAR5, OVCAR8, HEY, and 105C	- siRNA (DYRK1A and DYRK1B) - shRNA (p130, DYRK1A, p27kip1, and p57kip2)	Harmine * INDY *	N.D., but in vitro experiments showed that DYRK1A inhibition improved sensitivity to carboplatin	DREAM complex inhibition affected spheroid survival in ovarian cancer with carboplatin treatment	[25]
Cervical cancer	Caski, SiHa, and IC3	- Mutants (D6-10 or D21-24) of HPV16 E7 protein - shRNA (pRB, p107, and p130) - siRNA (BMYB) and overexpression (BMYB, FOXM1, wt/mutant E7, and LIN9), Flagged-16E7 ChIP-seq	-	N.D.	HPV viral protein E7 interacts with the BMYB-MuvB complex, inducing pocket protein degradation	[87]
	Hela and SiHa	LIN52 mutation (S20C)	-	N.D.	HPV16 E7 disrupts DREAM complex assembly	[88]
Meningioma	160 meningioma tumors from 140 patients and immortalized arachnoid cells	DNA/RNA sequencing of patient-derived primary tumors	-	Validation of the association with aggressive meningiomas using patient samples	Loss of DREAM complex repression is characteristic of aggressive meningiomas	[89]
Breast adenocarcinoma	MCF7, HCC1500, and PDX BC6 tumors, patient samples from those treated with neoadjuvant ribociclib in the FELINE phase 2 clinical trial (NCT02712723)	- p53 KO, MDM OE, etc. - CDK4/6i and CDK2i - Single-cell RNA sequencing of patient cohort samples	Abemaciclib ** or Ribociclib ** with AZD8421 (CDK2i)	CDK4/6i and CDK2i combination treatment improves the efficacy of CDK4/6i	Combination of CDK4/6i and CDK2i induces DREAM complex formation in p53-mutated ER+ breast cancer and increases the CDK4/6 response rate	[90]
Lung adenocarcinoma (LUAD)	LUAD cell lines and xenograft—A549, H23, H1792, and H358 Murine LUAD cell lines—KP836, 952, PDX, and TC241	p130 KO, shDYRKs, and LIN52-S28A	Harmine * Pita-vastatin ** Cyclosporin A **	DREAM target genes are correlated with poor survival in LUAD patients	PAF involvement in lung tumorigenesis via DREAM complex inhibition	[57]

* DREAM complex-disassembling agents, ** DREAM complex-inducing agents.
